# Meal frequency and incidence of type 2 diabetes: a prospective study

**DOI:** 10.1017/S0007114521003226

**Published:** 2022-07-28

**Authors:** Xiaowen Wang, Yonghua Hu, Li-Qiang Qin, Jia-Yi Dong

**Affiliations:** 1 Department of Epidemiology and Biostatistics, School of Public Health, Peking University Health Science Center, Beijing 100191, People’s Republic of China; 2 Public Health, Department of Social Medicine, Osaka University Graduate School of Medicine, Osaka 5650871, Japan; 3 Medical Informatics Center, Peking University Health Science Center, Beijing 100191, People’s Republic of China; 4 Department of Nutrition and Food Hygiene, School of Public Health, Soochow University, Suzhou 215000, People’s Republic of China

**Keywords:** Meal frequency, Type 2 diabetes, Prospective study, Chinese

## Abstract

Dietary habits play an important role in the development of obesity and type 2 diabetes. However, evidence on association between diet frequency and type 2 diabetes was limited and inconclusive. We aimed to examine the association between meal frequency and risk of type 2 diabetes. The cohort study used data from China Health and Retirement Longitudinal Study of 8874 community-dwelling people aged over 45 years. Participants were classified as eating two meals per day, three meals per day and four meals per day. Multiple Poisson regression models were used to examine risk of 4-year incident type 2 diabetes among people who ate more or less than three meals per day compared with people who ate three meals per day. We documented 706 type 2 diabetes cases during follow-up. After adjustment for known risk factors for type 2 diabetes, except for BMI, participants who ate four meals per day were at a lower risk of type 2 diabetes than those who ate three meals per day (relative risk(RR) = 0·73 (0·58, 0·92)). After further adjustment for baseline BMI, the association was slightly attenuated but remained statistically significant (RR = 0·76 (0·60, 0·97)). Subgroup analysis showed that the fully adjusted RR of type 2 diabetes for people eating four meals per day were 0·66 (0·48, 0·91) and 0·93 (0·65, 1·34) among those had a BMI < 25 and ≥ 25 kg/m^2^, respectively. Eating four meals per day, compared with eating three meals per day was associated with lower risk of developing type 2 diabetes in a Chinese population, particularly in those with a BMI < 25 kg/m^2^.

Dietary habits, including eating speed, breakfast skipping and meal frequency, have received increasing interest because of their potential impacts on human health, especially metabolic-related diseases^(1–4)^. For instance, a number of observational studies have shown an association between a fast eating speed and increased risk of obesity^(4)^ and type 2 diabetes^(5,6)^. Breakfast skipping was also associated with an increased risk of type 2 diabetes^(3)^.

Observational studies suggested that increased meal frequency was associated with a lower risk of obesity^(7)^ and improved lipid profiles^(8–10)^. One meta-analysis of randomised controlled trials (RCT) investigating the effects of meal frequency on weight loss and body composition found that increased meal frequency had beneficial effects on body composition, including reductions in fat mass and increases in fat-free mass^(11)^. Such evidence indicated that increased meal frequency may be helpful in diabetes prevention. However, another recent meta-analysis of RCT indicated that body weight and waist circumference might be affected by meal frequency, but not fat mass and energy intake^(12)^. Also, cohort studies examining the association between meal frequency and type 2 diabetes posed controversial results. Two prospective cohort studies reported the non-significant association between eating frequency and risk of type 2 diabetes^(13,14)^, while one study found an increased risk of type 2 diabetes in people eating four meals per day compared with those eating 1–3 meals per day^(15)^.

Of note, all the three cohort studies^(13–15)^ were performed in the USA, where the prevalence of overweight and obesity is high, and no evidence existed from Asian population on this topic. Therefore, the objective of this study was to examine the association between meal frequency and risk of type 2 diabetes in a Chinese population. We also performed stratified analyses by BMI to test whether BMI could modify the association.

## Methods

### Participants

**Fig. 1.**
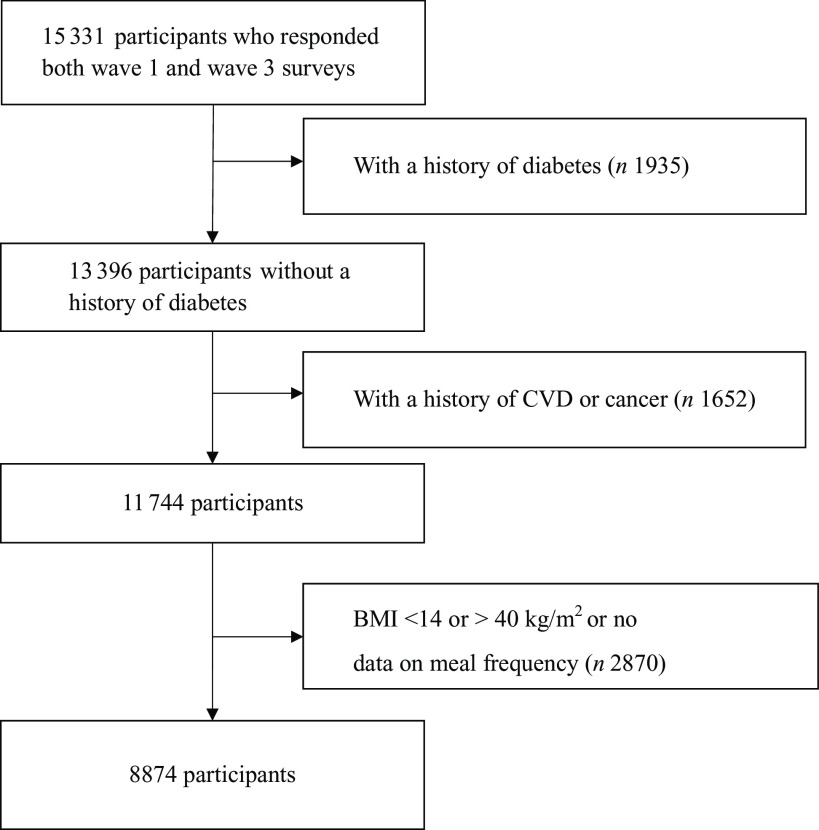
Flow chart of participant selection.

The study was based on a nationally representative cohort study derived from the China Health and Retirement Longitudinal Study (CHARLS). The design, sampling procedure, and data collection methods of the CHARLS have been previously described^(16)^. Briefly, CHARLS recruited residents aged over 45 years from 150 county-level units across 28 provinces in China. The baseline survey was conducted in 2011–2012 (wave 1) among 17 708 participants with a response rate of 80·5 %, and the study was followed up every 2 years. In wave 3 (2015–2016), 15 331 participants successfully responded. People with diabetes or high blood glucose at baseline were excluded: reporting a diagnosed history of diabetes, fasting blood glucose ≥ 126 mg/dl or HbA1c ≥ 6·5 %. We further excluded people who reported a history of heart disease, stroke or cancer. In addition, people with a BMI < 14 or > 40 kg/m^2^ or no data on meal frequency were further excluded. Finally, 8874 Chinese men and women were eligible for this analysis (Fig. 1).

All the participants completed a written informed consent before the survey. Ethics approval for CHARLS was obtained from the Biomedical Ethics Review Committee of Peking University (IRB00001052-11015).

### Assessment of meal frequency

Participants were asked about meal frequency by the question ‘how many meals do you normally eat every day’. Six options were provided: ‘more than 4 meals per day’, ‘4 meals per day’, ‘3 meals per day’, ‘2 meals per day’, ‘1 meal per day’ and ‘< 1 meal per day’. Because very limited people eating more than four meals per day or less than two meals per day, the main analysis was restricted in people eating 2–4 meals per day. In a sensitivity analysis, we divided the participants into three groups: ≤ 2 meals per day, 3 meals per day and ≥ 4 meals per day. In this questionnaire, meal frequency was defined based on main meals with staple food at customary or regular occasions. Snacks without staple food, such as beverages, desserts and chips, were not considered as a meal.

### Covariates measurement

Data on demographic background, physical and mental health status, and lifestyles were based on interviewer-administered questionnaires. Body weight, height and waist circumference were obtained from physical examination by well-trained clinicians. Participants erect on the floor board of the stadiometer and stand on scale, with shoes off. Waist circumference was measured by a soft measuring tape around waist, over clothing, holding securely at the level of navel. The BMI was calculated as weight (kg)/height (m)^2^. Venous blood samples were collected and measured for biomarkers, including fasting blood glucose, HbA1c and lipid profiles at the KingMed laboratory. The details of the assay used have been described previously^(17)^.

### Outcome assessment

Participants were considered as cases of type 2 diabetes if they were diagnosed to have diabetes by doctors, had fasting blood glucose ≥ 126 mg/dl or had HbA1c ≥ 6·5 % at wave 3 survey^(18–20)^. Validity of outcome assessment has been established elsewhere^(18–20)^.

### Statistical analyses

One-way ANOVA and Pearson χ^2^ tests were used to compare the mean values and percentages of characteristics at wave 1. To examine the association between meal frequency and type 2 diabetes risk, we estimated relative risks (RR) and 95 % CI by using the multiple Poisson regression models, which could be used to estimate the incidence of a disease among exposed and unexposed people^(21,22)^. People eating three meals per day were used as the reference group. Model 1 adjusted for age only. Model 2 further adjusted for sex, study area (urban *v*. rural areas), education level (primary school or lower, middle school, high school, college or higher education), marital status (married, divorced, widowed or never married), level of physical activity (0, 1–3, 4–6 and 7 d/week), history of hypertension (yes or no), history of dyslipidemia (yes or no), smoking status (never, past or current smoking) and drinking status (never drinking, past drinking, less than once/month or more than once/month). Baseline BMI was further adjusted in additional analysis. We further conducted stratified analyses according to baseline BMI (< 25 *v*. ≥ 25 kg/m^2^) to test whether the association could be modified by BMI. All analyses performed using SAS version 9.4 (SAS Institute Inc.). *P* values < 0·05 were considered statistically significant.

## Results

Table 1 shows the baseline characteristics of 8874 Chinese men and women according to their meal frequency. The numbers of participants who ate meals two times per day, three times per day and four times per day were 110 (1·2 %), 7574 (85·4 %) and 1190 (13·4 %), respectively. Compared with people who ate three meals per day, people eating four meals per day appeared to be slightly older and have a lower BMI and a lower waist circumference. Also, they were more likely to smoke and live in rural area but less likely to drink, be married, and have a higher education level and a history of hypertension. The baseline HbA1c, fasting blood glucose and lipid profile did not significantly differ by meal frequency (Table 1).

**Table 1.** Baseline characteristics of Chinese people according to meal frequency

	Two times per day (*n* 110)			Three times per day (*n* 7574)			Four times per day (*n* 1190)		
	%	Mean	sd	%	Mean	sd	%	Mean	sd
Age (year)		60·0	8·9		58·5	9·2		59·6	10·2
Men	53·4			49·0			48·5		
BMI (kg/m^2^)		22·1	3·4		23·2	3·4		22·6	3·4
Waist circumference (cm)		79·5	12·2		83·5	12·2		82·8	12·2
BMI ≥ 25 (kg/m^2^)	16·3			28·5			22·4		
Urban	16·4			34·2			23·4		
High school or higher education	6·5			10·3			6·3		
Married	83·8			89·1			82·6		
Hypertension	7·4			18·3			13·8		
Dyslipidemia	2·9			5·7			4·4		
Current smoker	30·1			32·2			37·6		
Current drinker	24·5			35·6			30·6		
Vagarious activity every day	47·7			25·2			24·9		
Moderate activity every day	60·1			47·2			40·3		
HbA1c (%)		5·12	0·5		5·10	0·5		5·06	0·5
Fasting blood glucose (mg/dl)		96	19		100	17		99	17
TAG (mg/dl)		105	51		118	53		115	50
TC (mg/dl)		193	37		193	38		189	38
LDL-cholesterol (mg/dl)		117	33		118	34		114	34
HDL-cholesterol (mg/dl)		55	14		52	15		51	15

TC, total cholesterol.

All values were means (standard deviation) or percentage.

We identified 706 cases of type 2 diabetes among 8874 Chinese men and women over 4 years of follow-up. As is shown in Table 2, compared with people who ate three meals per day, those eating four meals per day had a lower risk of developing type 2 diabetes in the age-adjusted model (RR = 0·73 (0·57,0·92)). After adjustment for sex, study area, education level, marital status, level of physical activity, history of hypertension, history of dyslipidemia, smoking status and drinking status, the association changed little (RR = 0·73 (0·58, 0·92)). After further adjustment for baseline BMI, the association was slightly attenuated but remained statistically significant (RR = 0·76 (0·60, 0·97)). Eating two meals per day was not associated with risk of type 2 diabetes in all models. In the sensitivity analysis with three groups (≤ 2, 3 and ≥ 4 meals per day) yielded similar results (online Supplementary Table 1).

**Table 2.** Association between meal frequency and type 2 diabetes

	Two times per day		Three times per day	Four times per day	
No. of cases	9		626	71	
	RR	95 % CI		RR	95 % CI
No. of participants	110		7574	1190	
RR, model 1	1·11	0·61, 2·02	1·00	0·73	0·57, 0·92
RR, model 2	1·17	0·64, 2·14	1·00	0·73	0·58, 0·92
RR, model 2 + BMI	1·25	0·68, 2·27	1·00	0·76	0·60, 0·97

RR, relative risk.

Model 1: Age.

Model 2: Further adjusted for sex, study area, highest education level, marital status, level of vagarious activity, level of moderate activity, history of hypertension, history of dyslipidemia, smoking status and drinking status.

We next conducted a stratified analysis by baseline BMI. People eating two meals per day were excluded from this analysis because of a small number in this group. The fully-adjusted RR of type 2 diabetes for people eating four meals per day were 0·66 (0·48, 0·91) and 0·93 (0·65, 1·34) among those had a BMI < 25 and ≥ 25 kg/m^2^, respectively (Table 3). Interaction tests suggested that baseline BMI did not modify the association between meal frequency and incidence of type 2 diabetes (*P*_interaction_ = 0·11).

**Table 3.** Association between meal frequency and type 2 diabetes by baseline BMI

		Four times per day		
	Three times per day	RR	95 % CI	*P*_interaction_
Baseline BMI < 25 (kg/m^2^)				
No. of cases	342	38		
No. of participants	5407	930		
RR, model 1	1·00	0·65	0·47, 0·90	0·13
RR, model 2	1·00	0·64	0·47, 0·89	0·07
RR, model 2 + BMI	1·00	0·66	0·48, 0·91	0·11
Baseline BMI ≥ 25 (kg/m^2^)				
No. of cases	284	33		
No. of participants	2167	260		
RR, model 1	1·00	0·95	0·67, 1·36	
RR, model 2	1·00	0·94	0·66, 1·35	
RR, model 2 + BMI	1·00	0·93	0·65, 1·34	

RR, relative risk.

Model 1: Age.

Model 2: Further adjusted for sex, study area, highest education level, marital status, level of vagarious activity, level of moderate activity, history of hypertension, history of dyslipidemia, smoking status and drinking status.

## Discussion

In this prospective study of Chinese people, we observed that eating four meals per day, compared with eating three meals per day, was associated with lower risk of developing type 2 diabetes. This association was evident among people with a BMI < 25 kg/m^2^ but was non-significant among people with a BMI ≥ 25 kg/m^2^. Eating two meals per day was not associated with type 2 diabetes.

Potential mechanisms underlying the association between meal frequency and the risk of type 2 diabetes warrant discussion. Several lines of evidence from cross-sectional studies have suggested an inverse association between increased eating frequency and prevalence of obesity^(7)^. Specifically, there was evidence that four meals per day compared with three meals per day was associated with a lower prevalence of general and central obesity^(23,24)^. Furthermore, a RCT found an increase in fat mass among young lean male participants who switches from four meals per day to three meals per day (the usual afternoon meal was skipped)^(25)^. In addition, higher eating frequency may also improve blood lipid concentrations. A recent meta-analysis of twenty-one RCT suggested that higher meal frequency may help improve total cholesterol and low-density lipoprotein cholesterol^(26)^, though blood glucose was not affected. In a subgroup analysis of trials > 12 weeks, higher meal frequency may also reduce serum TAG and increase HDL-cholesterol. Overall, eating frequency may be one of the contributors affecting body weight, fat deposition and serum lipid concentrations, which involves in the pathogenesis of type 2 diabetes^(27)^.

To our knowledge, only three prospective cohort studies^(13–15)^ have reported the association between meal frequency and risk of type 2 diabetes. The Nurses’ Health Study (NHS) of US women showed that in comparison with women who ate three times per day, the RR for type 2 diabetes were 1·09 (0·84, 1·41) for women who ate 1–2 times per day and 1·13 (1·00, 1·27) for women who ate 4–5 times per day. However, after further adjusting for baseline BMI, no significant association remained (RR: 1·13 (0·87, 1·46) and 1·04 (0·92, 1·17), respectively)^(13)^. The Health Professionals Follow-Up Study (HPFS) of US men demonstrated that participants who ate 1–2 times per day or four times per day were at higher risk of type 2 diabetes than those who ate three times per day (RR: 1·26 (1·09, 1·46) and 1·19 (1·07, 1·32), respectively). After further adjustment for BMI, the risk for men who ate 4–5 times per day became non-significant (RR: 1·09 (0·99, 1·21))^(14)^. Another cohort study from the Women’s Health Initiative Dietary Modification Trial (WHI-DM) showed similar results that four meals per day compared with 1–3 meals per day was associated with an increased risk of type 2 diabetes in postmenopausal women (HR: 1·38 (1·03, 1·84))^(15)^. Overall, these three cohort studies of US populations showed an increase in risk of type 2 diabetes among people eating four or five times per day, which may be partly explained by baseline BMI.

Of note, the above three studies were all conducted in the USA, where the prevalence of overweight and obesity is high. The baseline BMI in US adults was substantially greater than that observed in our study. Our study included lean participants with a mean BMI ranging from 22 to 23 kg/m^2^ across groups, whereas the mean BMI in the US studies was greater than 25 kg/m^2^ (and even >28 kg/m^2^ in WHI-DM study). Our subgroup analysis observed no significant association among those with a BMI ≥ 25 kg/m^2^, which was in line with the results from NHS and HPFS study. Participants with a higher BMI may have chronic inflammation, dyslipidemia, impaired postprandial metabolism or insulin resistance^(28,29)^. These metabolic disorders may attenuate the potentially beneficial effects of a higher meal frequency. Moreover, the questionnaires about eating frequency in the US studies included meals and snacks, while in our study, only meals with staple food were considered as diet frequency. There was evidence that additional snacks beyond the main meals were associated with type 2 diabetes risk^(14,30)^. Taken together, differences in participant baseline BMI and diet frequency measurements may be responsible for the discrepant results between this study and previous ones.

Our study was not powered to evaluate the association of eating two meals per day with type 2 diabetes due to a small number of participants in this group. A 23-year cohort study demonstrated that skipping meals to lose weight was associated with higher risk of type 2 diabetes among men, elevated cholesterol, high blood pressure and restless sleep^(31)^. In addition, emerging evidence suggested that skipping breakfast was associated with increased risks of type 2 diabetes and CVD^(3,32)^, probably by maintaining a healthy weight and lipid profile^(33)^. Unfortunately, we had no data on which meal was skipped and could, therefore, not examine the association for breakfast skipping.

This is the first prospective study that showed an inverse association of meal frequency with the risk of type 2 diabetes incidence. We used nationally representative data and a prospective cohort design. However, limitations of the present study should be acknowledged. First, the CHARLS study did not conduct dietary assessment and we were therefore unable to control for food, nutrient and total energy intakes. Although a high-quality diet was associated with lower risk of type 2 diabetes^(34)^, evidence on association between meal frequency and diet quality was inconsistent^(35–37)^. Furthermore, a recent meta-analysis of RCT also found that no significant effects were observed on energy intake comparing the different meal frequencies^(12)^. In line with these reports, the NHS study showed that further adjustment for total energy intake, cereal fibre intake and the Alternative Health Eating Index 2010 changed the results little^(13)^. Nevertheless, residual confounding due to dietary intakes (e.g. snacks) and other unmeasured risk factors (e.g. family history of diabetes) remained a possible explanation for the observed association. For example, snacks are usually fatty, sugary, salt and fried foods with low nutrient profiles and high in energies, which are associated with weight gain and higher risk of type 2 diabetes^(14,30,38)^. Thus, it might bias the association towards a higher risk without adjusting snacks. Second, the validity of meal frequency has not been carried out. The self-report dietary information was subject to misreporting and measurement error. However, such measurement error was likely to be non-differential in the prospective study and led to associations being biased towards the null. Moreover, lifestyle changes (e.g. smoking, drinking status changes) could also have an impact on the outcomes. Third, our observations were conducted in a Chinese population, caution should be paid to generalise the results to other populations with different genetic backgrounds, diet habits and lifestyles.

In conclusion, eating four meals per day, compared with eating three meals per day, was associated with lower risk of developing type 2 diabetes in a Chinese population, particularly in those with a BMI < 25kg/m^2^. The results from our study suggested that meal frequency might be a potential modifiable factor for type 2 diabetes and having an appropriate meal frequency may have a role in the prevention of type 2 diabetes.

## Supplementary material

For supplementary material accompanying this paper visit https://doi.org/10.1017/S0007114521003226. click here to view supplementary material
