# Supplementary material for: Meal frequency and incidence of type 2 diabetes: a prospective study
Source: Br J Nutr. 2021 Aug 23;128(2):273–8. doi: 10.1017/S0007114521003226 (PMC9301526; doi:10.1017/S0007114521003226)
Supplement: Supplementary file 1 [file S0007114521003226sup001.docx]

Supplementary Table 1. Association between meal frequency and type 2 diabetes

|  | ≤ Twice/day | Three times/day | ≥ Four times/day |
| --- | --- | --- | --- |
| No. of Cases | 19 | 626 | 76 |
| No. of participants | 226 | 7574 | 1208 |
| RR, model 1 | 0.94 (0.60, 1.49) | 1.00 | 0.72 (0.57, 0.91) |
| RR, model 2 | 1.04 (0.64, 1.69) | 1.00 | 0.72 (0.57, 0.92) |
| RR, model 2 + BMI | 1.13 (0.70, 1.84) | 1.00 | 0.75 (0.59, 0.96) |

Model 1: age

Model 2: further adjusted for sex, study area, highest education level, marital status, level of vagarious activity, level of moderate activity, history of hypertension, history of dyslipidemia, smoking status, and drinking status.

Abbreviation: RR: relative risk; BMI: body mass index
